# A tuberculosis outbreak at an insecure, temporary housing facility, manga café, Tokyo, Japan, 2016–2017

**DOI:** 10.1017/S0950268819001092

**Published:** 2019-06-26

**Authors:** M. Endo, M. Ota, A. Kayebeta, I. Takahashi, Y. Nagata

**Affiliations:** 1 Shinjuku City Health Office, Tokyo, Japan; 2 Research Institute of Tuberculosis, Tokyo, Japan

**Keywords:** Contact investigation, disease outbreak, epidemiology, tuberculosis

## Abstract

In November 2016, a woman in her 30s who stayed at an insecure, temporary housing facility, a manga café in Tokyo, Japan, for a year was diagnosed with sputum smear-positive tuberculosis (TB). Since the café had 31 staff members and provided with accommodation to many people, the local health office initiated a contact investigation. This study aims to characterise the cases found in the outbreak. A TB case was defined as a person tested bacteriologically positive for TB, or was determined to have TB by a physician. A latent TB infection case was defined as a person tested positive by interferon-*γ* release assay. From January 2016 through November 2017, there were 31 staff members at the manga café, of which, six developed TB disease (one smear-negative, culture-positive and five smear- and culture-negative) in addition to seven LTBI. Another long-term customer was found having sputum smear-positive TB. Variable numbers tandem repeat (VNTR) test revealed that the index patient and the long-term customer had the identical type of VNTR; however, one staff member had a different VNTR. Local health authorities should intensify screening long-term customers of such facilities for TB regularly as well as once a TB outbreak occurs.

## Introduction

In Japan, the tuberculosis (TB) notification rate has declined in the past six decades from 698.4 per 1 00 000 population in 1951 to 17.7 per 1 00 000 population in 2011 [1]. However, about 8000 smear-positive TB cases are still reported annually [2], and these infectious TB cases pose a public health threat to the community. TB outbreaks have also been reported, involving hospitals, workplaces, schools and sometimes homeless people [3–9]; however, only a few involving homeless people were reported in the past decade.

Shinjuku is a special ward in Tokyo, Japan. It is a major commercial and administrative centre, housing the northern half of the busiest railway station in the world (Shinjuku Station) and the Tokyo Metropolitan Government Building, the administration centre for the government of Tokyo. As of 2015, the ward has an estimated population of 338 000, and a population density of 18 500 people per square kilometres. The total area is 18 km^2^. Since the end of the World War II, Shinjuku has been a major secondary centre of Tokyo [10]. The population of homeless persons in Shinjuku was estimated to be 99 in August 2015 [11], decreased from 299 in January 2009 [12]. TB notification rate for all types of TB in Shinjuku was 33.7 per 100 000 population in 2016, which has been 2.4 times as high as that of the entire country with two peaks in young (66.5 per 100 000 population in those aged 20–29 years) and in elderly (68.0 per 100 000 population in those aged 80 years or older). Meanwhile, treatment outcomes were relatively good (88.2% for treatment success for all types of TB for the 2015 cohort) with its intensive DOT programme.

In November 2016, a woman in her 30s who had stayed at an insecure, temporary and low-price housing facility, the so called manga (comic books) café [13], near Shinjuku Station for about a year was diagnosed with sputum smear-positive pulmonary TB (patient 1). Although she had had a cough, excretion of sputum, low-grade fever and dyspnoea for a couple of months, she had not sought healthcare and had not been diagnosed. Although such cafés, including net cafés, originally provided only Internet or manga library services, some have expanded their services to include food, drink and showers, and are open 24 h a day, 7 days a week. They are often used by commuters who miss the last train; however, the net café refugees’ trend has seen large numbers of people used them as their homes [14]. Since the manga café provided with accommodation to over 100 people and had about 30 staff members, the local health office initiated a contact investigation.

The practice of contact investigations of TB contacts in Japan [1, 5, 7, 15] is similar to that recommended elsewhere [16], however, interferon-*γ* release assay (IGRA) [17, 18], rather than tuberculin skin testing (TST), is commonly used [19, 20] to screen latent TB infection (LTBI), because IGRA is more specific and can avoid interference caused by Bacillus Calmette–Guérin (BCG) vaccination.

This study aims to characterise the cases found in the TB outbreak and to call for a greater attention to TB risk in the populations involving insecure, temporary housing facilities.

## Methods

A TB case was defined as a staff member or a customer at the manga café who had bacteriologically positive TB in the sputum sample determined by smear microscopy, culture or nucleic acid amplification, or was determined to have TB by a physician via a chest X-ray, a chest computed tomography (CT) scan, histological or pathological tests, or clinically from January 2016 through December 2017. However, a TB case who had a different strain of *Mycobacterium tuberculosis (MTB)* than the dominant strain was excluded from the cases and thus further analysis, because it was likely that the case was infected with TB from a different source than the current event. An LTBI case was defined as the one in which a person tested positive in an IGRA test during the same period. However, the one who had a history of IGRA positivity was excluded from the analysis, because it is not possible to attribute the IGRA positivity to the current event.

This is a cohort study and all the staff members who worked at least one day and the manga café customers who stayed more than 8 h during patient 1's infectious period that was determined as from September to November 2016 were enrolled as the contacts. We also searched the routine surveillance data for recently diagnosed TB patients who stayed at the manga café before the diagnosis.

The information related to the demographic characteristics and the work shift of the staff members and the length of stay of the manga café customers was collected from the staff members or the administrative files at the manga café.

The contacts were tested with IGRA in January to March 2017. All the IGRA-positive contacts were referred to a chest physician who was familiar with TB for further investigation, including chest X-ray and CT scan tests, if indicated. The persons who had abnormal findings suggestive of TB were put on the standard anti-TB treatment. Those who were IGRA-positive without any abnormal shadows in the chest X-ray or CT scan were persuaded to go on LTBI treatment with isoniazid for 6–9 months.

All the isolates of *MTB* derived from the cases were analysed on the variable number of tandem repeats (VNTR) in 24 loci for comparison by Tokyo Metropolitan Institute of Public Health with methods recommended by Iwamoto *et al*. [21].

Analysis of the IGRA positivity using a Fisher's exact test with a calculation of 95% confidence intervals was carried out with the R (The R Foundation, Vienna, Austria). A *P*-value <0.05 was considered statistically significant.

## Results

There were 31 staff members of the manga café during patient 1's infectious period (Table 1). All of them were Japanese-born. Six (19.3%) out of the 31 staff members developed TB disease (patient 2 through 7), one of whom was smear-negative and culture-positive (patient 3) and the remaining five were smear- and culture-negative with findings suggestive of TB in the CT images without any signs and symptoms (four were IGRA-positive and one negative); however, patient 3 had a different strain of *MTB* than the others, as mentioned later, the patient was excluded from further analysis. Seven (22.6%) additional staff members had LTBI. An additional staff member had been known to be positive for IGRA for 4 years, because she had had a close contact with a TB case, and she was excluded from further analysis.

**Table 1.** Characteristics of the staff members and the long-term customers investigated in relation to a tuberculosis outbreak at a manga café, Tokyo, Japan, 2016–2017

	Staff members	Long-term customers
Number	31	4^a^
Age (median)	25 (range: 19–36)	39.5 (range: 23–58)
Sex	M:F = 19:12	M:F = 3:1
Working days/week		
5 days	5	n/a
3–5	15	n/a
<3 days	11	n/a
Length of stay		
3 months	n/a	4

n/a, not applicable.

a The number includes one who was found having TB from the surveillance data.

Regarding the customers of the manga café, the gross number of the customers during patient 1's infectious period was over 2000, however, most customers were unable to be specified because those who use manga library or who just stay (or live) there did not have to identify themselves: only those who use the Internet have to produce identity cards under the local regulations. The staff members were only able to specify 11 very long-term customers who possessed a large amount of belongings in their cubicles. The local health office staff members tried their best to persuade them to take health examinations, however, out of which, only three agreed. All of the three long-term customers had stayed at the manga café for more than 3 months during patient 1's infectious period; however, none had either TB disease or LTBI.

From the surveillance data, it was found that one long-term customer of the manga café in his 50s had been diagnosed as sputum smear-positive pulmonary TB in September 2016 (patient 8). He had lived at the café consecutively for over 4 years before he was diagnosed and hospitalised. He had cough since June that year and diabetes mellitus for years; however, he did not seek health care before the diagnosis. Upon recognition of patient 8, the local health office re-evaluated the potential contacts. The staff members who had contact with patient 8 during his infectious period determined as from June through September 2016 were the same.

**Fig. 1.**
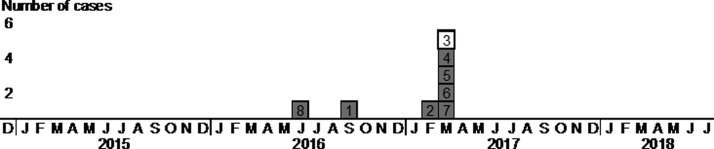
Epidemic curve of tuberculosis cases in relation to a manga café outbreak, Tokyo, Japan, 2016–2017. A box indicates the timing of the development of symptoms or diagnosis (if asymptomatic) of the tuberculosis patients. The numbers in the boxes are the patients' ID numbers referred in the main text. It was later found that patient 3 had a different strain of *Mycobacterium tuberculosis* than that of patients 1 and 8 who were considered as source cases.

**Fig. 2.**
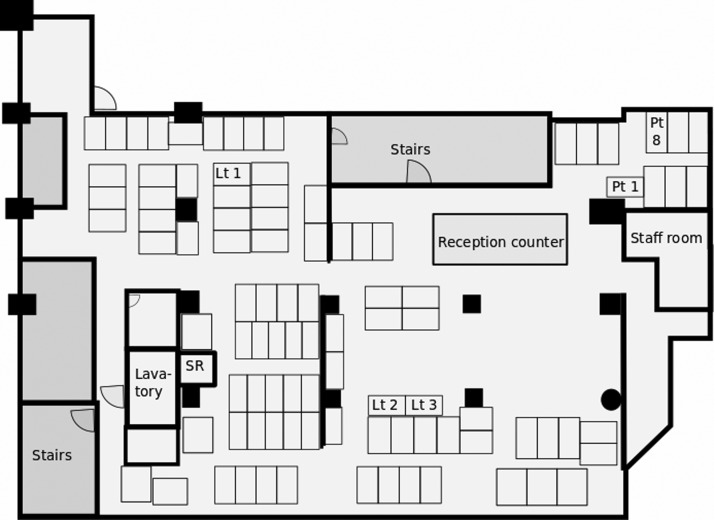
Schematic map of the manga café in relation to a tuberculosis outbreak, Tokyo, Japan, 2016–2017. The small thin blank boxes represent cubicles of customers. Lt 1–3 = The cubicles of the long-term customers who were investigated; PT1, 8 = the cubicles of the TB patients who were also long-term customers. Patient 1 was diagnosed in November and patient 8 in September 2016. SR, shower room.

An epidemic curve of TB patients found in the outbreak is shown in Figure 1. Since the screening and further examinations for the staff members were conducted in January to March 2017, most secondary cases were found in these months. The schematic map of the manga café is shown in Figure 2. There were over 100 cubicles and a reception area, whose airspaces were not physically separated from each other. The staff room where the staff members took rest was physically separated from the other areas, however, the airspace was not isolated; the air was circulated to and from the other areas. The cubicles of patients 1 and 8 were very close to each other, located just across an aisle. The reception area was also close to the cubicles of patients 1 and 8. The other very long-term customers' cubicles were away from that of patients 1 and 8.

Table 2 gives the numbers and the proportions of the staff members and the very long-term customers with TB disease, LTBI and TB disease plus LTBI. The data for the staff members were disaggregated by the working days per week. There was no statistically significant difference in the proportion of either TB disease, LTBI or TB disease plus LTBI by the working days per week.

**Table 2.** Numbers of tuberculosis cases and latent tuberculosis cases among the staff members and the long-term customers in relation to tuberculosis outbreak at a manga café, Tokyo, Japan, 2016–2017

	Tuberculosis (TB) disease			Latent TB infection			TB disease and latent TB infection					Total
	*n*	%	95% CI	*n*	%	95% CI	*n*	%	95% CI	RR	95% CI	
Staff members												
worked 5 days/week	0	0.0	0–52.2	3	60.0	14.7–94.7	3	60.0	14.7–94.7	2.4	0.38–11.3	5
3–5 days/week^a^^,^^b^	3	23.1	5.0–53.8	3	23.1	5.0–53.8	6	46.2	19.2–74.9	1.8	0.31–11.1	13
<3 days/week	2	18.2	2.3–51.8	1	9.1	0.23–41.3	3	27.3	6.0–61.0	1.1	0.15–7.7	11
Subtotal	5	17.2	5.8–35.8	7	24.1	10.3–43.5	12	41.4	23.5–61.0	1.7	0.29–9.5	29
Long-term customers^c^	1	25.0	0.63–80.6	0	0.0	0–60.2	1	25.0	0.63–80.6	1.0	–	4
Total	6	18.2	7.0–5.5	7	21.2	9.0–8.9	13	39.4	22.9–57.9	–	–	33

TB, tuberculosis; LTBI, latent TB infection; RR, risk ratio; CI, confidence interval.

a Excluding the TB case with a different strain of *Mycobacterium tuberculosis* than the other patients.

b Excluding the known LTBI case.

c Excluding the index patient.

Table 3 shows the results for 24 loci of VNTR for three culture-positive TB patients (patients 1, 3 and 8). The VNTR results were identical for patients 1 and 8, however, that of patient 3 (staff member) was different from the other two.

**Table 3.** The results of variable number tandem repeats (VNTR) test of patients found in a tuberculosis outbreak at a manga café, Tokyo, Japan, 2016–2017

Loci	1	2	3	4	5	6	7	8	9	10	11	12	13	14	15	16	17	18	19	20	21	22	23	24
Pt 1	4	3	4	3	6	3	7	4	5	7	8	3	8	8	4	14	12	10	3	3	2	4	3	4
Pt 3	3	3	3	4	7	3	7	5	5	7	2	5	10	8	4	12	12	11	3	3	2	4	4	4
Pt 8	4	3	4	3	6	3	7	4	5	7	8	3	8	8	4	14	12	10	3	3	2	4	3	4

Pt 1: A long-term customer with tuberculosis disease who was diagnosed in September 2016.

Pt 3: A staff member with tuberculosis disease who was diagnosed in March 2017.

Pt 8: A long-term customer with tuberculosis disease who was diagnosed in November 2016.

## Discussion

We conducted a TB outbreak investigation at a manga café in Tokyo and found seven TB patients, in addition to the index TB patient, among the staff members and the long-term customers as well as seven LTBI patients among the staff members. Over 40% of the staff members either developed TB disease or were infected with TB. One patient of the long-term customers had an identical strain of *MTB* with the index patient; however, another patient of the staff members had a different strain of *MTB*.

The reason why the two patients of the long-term customers had the identical strain of *MTB* was because their cubicles were very close to each other and they stayed at the café more than a year, and thus one of the two, most probably patient 8, considering the sequence of development of the symptoms, spread TB to the other. Either one of the two patients, or both, also spread TB to the staff members, except patient 3, who worked at the reception area or rested in the staff room, both of which were also close to the cubicles of patients 1 and 8, because sputum smear-positive TB patients, particularly super spreader ones, are much more likely to spread TB to others than smear-negative, culture-positive patients [22–25]. The other three very long-term customers were not infected with TB, probably because their cubicles were away from that of patients 1 and 8.

There were a few reports on TB outbreaks involving net cafés or saunas (bathhouses) in Japan in late 1990s [9, 26]. The situations were quite similar with the current event: indigent sputum smear-positive TB patients who stayed at the cafés or saunas spread TB to staff members and other long-term customers. They had had symptoms for long before diagnosis but did not seek health care. Thus, these insecure, temporary housing facilities could become the hotspots for TB infection. In other parts of the world, similar findings were observed. A single highly infectious index case in Canada who was symptomatic for long before diagnosis and who stayed at a homeless shelter spread TB to the worker and the volunteers at the shelter or the other persons with a history of homelessness [27]. In the USA, 28 outbreak-associated TB cases were reported involving only homeless shelter guests, but shelter workers, from 2007 to 2011, probably because the transmission may have occurred outside of the shelter as well [28].

Our study has strengths and limitations. We were able to conduct descriptive as well as analytic epidemiology on TB and LTBI patients in relation to a TB outbreak at the manga café. Besides, we utilised IGRAs, rather than TST, for screening LTBI cases and the results were more accurate than when TST was utilised. However, we were able to examine only three out of 11 very long-term customers of the café, were not able to examine many other long- and short-term customers and thus we were not able to evaluate the risk of TB infection among the customers, because the manga café did not maintain most of the contact information of the customers. This is a major limitation of the study. Considering the situation in which unspecified, a large number of customers used the café and the majority of the long-term customers were not willing to cooperate with the local health staff and/or had difficulty understanding why they should undergo TB screening possibly because of depression or other psychiatric challenges [29, 30], we believe this was very difficult to manage. Another limitation was that we did not conduct IGRA tests for the staff members right after patient 1 was diagnosed in November 2016 and were not able to show IGRA conversion of the seven staff members with LTBI. However, since patient 1's infectious period was 3 months, we considered most staff members with LTBI must have converted to IGRA-positive when patient 1 was diagnosed. In addition, because IGRA-positivity of young Japanese-born population is very low (0.85–1.0% [15, 19, 20]), it may not be necessary to show IGRA conversion for young Japanese contacts: we considered most staff members with IGRA-positivity had been infected during the outbreak, not before. Third, we were not able to link the two long-term customers and the staff members with TB in molecular epidemiology and actually one of the staff members had a different stain than the two long-term customers. Since the six staff members with TB were found during the contact investigation, it is not unusual that almost all were at a very early stage of the disease, bacteriologically negative and clinically diagnosed. Thus, this limitation is practically inevitable. There is a slight possibility that a few of the staff members may have been infected with different strains of *MTB*, possibly because working in such an environment may be at high risk of being infected with TB. However, considering the timing of the development of TB of the staff members and the fact that two long-term customers had sputum smear-positive TB and had respiratory symptoms for some time before the diagnoses, we believe almost all, it might not be all, were infected with the same strain of the long-term customers rather than the staff member with TB (i.e. patient 3) who was smear-negative and culture-positive.

One of the recommendations is that the local health office makes more efforts to enrol and screen as many long-term customers of insecure, temporary housing facilities as possible for TB regularly and when a TB outbreak occurs, as long-term customers of such facilities are at high risk of TB. The local health office has been offering TB screening for 2 days per year for long-term customers of such cafés. With the aid of local non-governmental organisations helping vulnerable and/or marginalised population and peer advocates, and with the training of health care workers, a better uptake of TB screening could be possible. The staff members of an insecure, temporary housing facility, including Internet and manga cafés, and saunas, should regularly undergo TB screening and chest X-ray, if indicated, because they are at higher risk of TB infection based on our and other findings [9, 26]. The local health office has been offering health screening for free to such facilities for years; however, so far it has not been very successful. Another recommendation is that *MTB* strains cultured from local TB patients should regularly be collected and VNTR analysis should routinely be conducted on the strains to further understand TB epidemiology in this kind of congested areas, because it is quite likely that the *MTB* strain of patients 1 and 8 or other popular strains may come back from time to time among such underserved population in Shinjuku area.
